# Strengths and Pitfalls of Meta-Analysis Reports in Vesicoureteral Reflux

**DOI:** 10.1155/2008/295492

**Published:** 2008-05-16

**Authors:** K. Afshar, A. E. MacNeily

**Affiliations:** ^1^Department of Urologic Sciences, University of British Columbia and BC Children's Hospital, Vancouver BC, Canada V5Z 1M9; ^2^Department of Health Care and Epidemiology, University of British Columbia, Vancouver BC, Canada V6T 1Z3

## Abstract

There are many ongoing controversies surrounding vesicoureteral reflux (VUR). These include variable aspects of this common congenital anomaly. Lack of evidence-based recommendations has prolonged the debate. Systematic reviews (SRs) and meta-analysis (MA) are considered high-level evidence. The purpose of this review article is to summarize and critically appraise the available SR/MA pertaining to VUR. We also discuss the strength and pitfalls of SR/MA in general. A thorough literature search identified 9 SRs/MAs relevant to VUR. Both authors critically reviewed these articles for contents and methodological issues. There are many concerns about the quality of the studies included in these SRs. Clinical heterogeneity stemming from different patient selection criteria, interventions, and outcome definitions is a major issue. In spite of major advances in understanding different aspects of VUR in the last few decades, there is a paucity of randomized controlled trials in this field.

## 1. INTRODUCTION

Vesicoureteral reflux (VUR) is one of the most controversial topics in pediatric urology. The debate entails several aspects of VUR, such as clinical significance, diagnosis, treatment options and outcomes. The advent of endoscopic treatment of VUR has added to the complexity of this debate.

In an era of evidence-based medicine, there is a constant drive to use best available evidence in every day practice. Although systematic review (SR) and meta-analysis (MA) are well-established methods in generating evidence-based statements, they are not flawless. Specific steps should be taken to perform SRs, and clinical or statistical judgment calls are required of the authors. In addition, the quality of the available studies has a direct impact on the quality of SRs.

## 2. METHODS

In this review article, we first briefly explain the steps of a well-performed SR/MA [1] and then apply them to the topic of VUR. We did not intend to perform a systematic review of the topic but rather summarize and discuss the available SR/MA. Therefore, although we performed a thorough literature search, we did not use a conventional SR protocol. We included all the available SR/MA discussing any aspect of VUR (screening, diagnosis, or treatment). Both authors reviewed and critically appraised all articles.

**What is a systematic review (SR) and meta-analysis (MA)?**

SR is a method for secondary data analysis. In these types of studies, the authors attempt to identify all of the completed studies in relation to a specific research question in a systematic predefined manner. Then by using statistical methods, these results are combined to answer the research question based on all eligible studies. The actual statistical component of a systematic review is referred to as the MA.

**Steps in SR/MA**

1. *The research question*: the cornerstone of an SR/MA must be a clear and specific question(s).
2. *Definition of inclusion and exclusion criteria*: for example the authors may only include randomized controlled studies. Types of intervention(s), study population(s), and outcomes of interest should all be clearly stated. At least two authors should assess studies eligibility.
3. *Unbiased identification of all completed studies*: it is of paramount importance that a reproducible protocol be defined for the identification of studies. This includes a search of all available databases, a hand search of references and conference proceedings, and contact of experts in the field of interest. One common pitfall is to limit the search to few words or a single database such as MEDLINE. A medical librarian is an invaluable resource in developing effective search strategies.
4. *Collection of data from each study*: standardized data collection forms are available and should be used to facilitate the subsequent analysis. Quality of the included studies is also assessed and recorded. There are multiple validated tools for this purpose. It is recommended that at least two authors collect the data independently.
5. *Clear presentation of findings*: a summary of the results of literature search and reasons for exclusion of studies should be provided. Quality of the included studies should be discussed. The quality of reporting of meta-analyses (QUOROM) statement provides valuable guidelines for the authors [2].
6. *The process of meta-analysis*
  - Summary effect estimate and confidence interval. This is an average effect size, weighted by the size of the study. For example if the meta-analysis is combining the effect of a procedure versus antibiotics in preventing UTI, the final effect size is presented by a relative risk (RR). This is a weighted average size and describes a ratio between the incidence of UTI in two groups. The 95% confidence interval determines the statistical significance of the summary effect measure. If the interval is including 1 (RR = 1: equal incidence of UTI in the 2 groups), the findings are not statistically significant (*P* > .05).
  - Heterogeneity: if the studies are too heterogeneous in terms of design, population, intervention or outcomes, they should not be combined. The authors must decide about this important issue based on their expertise and judgment. Combining these dissimilar studies will lead to clinical heterogeneity. There are statistical tests for assessing heterogeneity. If the *P* value of these tests is over 0.1, heterogeneity is unlikely, and combining the findings from the studies is reasonable. The *P* value of 0.1 is usually used instead of 0.05, to be less conservative in detecting heterogeneity. Forrest plot is a well-accepted graphic method to summarize the finding of M/A. It shows the effect size for each study and the whole analysis, along with the 95% CI. The results of the tests of heterogeneity and the sample size are usually presented as well.
  - Assessment of publication bias: it is not unusual that small and negative studies are not published. An easy way to detect this bias is to generate a funnel plot. This is a scatter plot with the measure of effect and sample size on the *X* and *Y* axis, respectively. If publication bias is present the portion of the graph representing negative studies will be lacking.
  - Subgroup and sensitivity analyses: in subgroup analysis the data from some subsets of studies are analyzed together (e.g., studies only looking at certain age groups are combined). In sensitivity analysis, the MA is done with and without certain studies to estimate their overall effects on the results.

**Figure 1:**
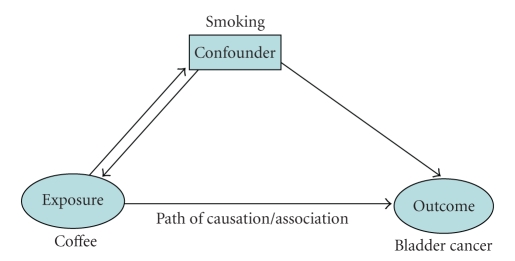
Confounder.

The major shortcoming of an SR is that its quality is totally dependent on the quality of the included studies, the socalled “garbage in-garbage out” effect. This is a major issue when observational studies are analyzed. Confounders and bias are the two main pitfalls of these types of studies. Confounders are factors associated with both exposure and outcome that are not in the path of causation (see Figure 1). For example, if a cohort study determines coffee drinking is associated with bladder cancer, one could consider cigarette smoking as a confounder. Smokers may drink more coffee (association with exposure). In addition, smoking is a known risk factor for bladder cancer (association with the outcome). So any association between consuming coffee and bladder cancer could be entirely due to the confounding effect of smoking. Bias is a systematic error in selection of cases, measurement of outcome, or analysis of the data. There are statistical ways to minimize confounding and bias but the most effective method is to randomize the participants. Therefore, the quality of individual studies should be assessed carefully and taken into account when interpreting the overall results of an SR/MA.

## 3. RESULTS

**Systematic reviews and meta-analysis for VUR:**

A thorough search of available literature yields 9 SR/MA pertaining to VUR. In the following paragraphs, we critically appraise the findings of each paper.

Shanon and Feldman published a review article in 1990 evaluating the methodology of studies on different aspects of VUR [3]. The article by no means fulfills the criteria of a modern SR due the lack of a reproducible protocol. They identified four subsets of article addressing the following facets of VUR: diagnosis, treatment, association with hypertension, and end stage renal disease. They concluded that VCUG is the gold standard for diagnosis of VUR. The 4 then available articles about treatment did not show any advantage for surgery compared to medical treatment in terms of preventing UTIs or renal scarring. The authors also concluded that although there is a possible association between VUR and hypertension or end stage renal disease, because of the low quality of the literature, it could not be estimated quantitatively. Although the conclusions of this review are of limited value today, this publication is of importance since it was the first attempt to critically appraise the VUR literature.

In an SR/MA, Gordon et al. reviewed the literature to answer the question: “Does the presence of VUR predict renal damage in children admitted to hospital with urinary tract infection (UTI)?” [4]. The authors identified 12 studies after screening 838 publications which were extracted from 3 major electronic databases. Screened studies were excluded if more than 10% of data was missing or if they dealt with outpatients. Test of homogeneity revealed significant heterogeneity among the included studies. This is partly related to different patient populations and study protocols. Subgroup analysis was not performed. The authors concluded that the presence of VUR is a weak predictor of renal damage, since a positive voiding cysto-urethrogram (VCUG) only increased the chance of a positive DMSA renal scan by 20% and a negative VCUG reduced this chance by 8%.

Although this SR/MA utilized sound methodologies, there are some important shortcomings. Above all, the authors do not discuss the type and quality of the studies included. It is not clear to the reader if these studies were prospective or retrospective. Retrospective studies are prone to bias and confounding and generally are less valid. Exposure and outcome definitions may not be the same. For example how was the UTI diagnosed? what constitutes a positive DMSA scan? All these factors contribute to the significant heterogeneity and make the interpretation more difficult. In addition, the findings are not widely generalizable since this SR only included inpatients in an era when most UTIs are managed as outpatients.

Wheeler et al. published an SR/MA regarding antibiotics versus surgery for the treatment of VUR [5]. The authors only included randomized controlled trials (RCTs) which allowed the analysis of 8 studies involving 859 children. No significant difference was found in terms of renal scars and recurrence of a febrile UTI between the two groups. Nevertheless, children treated with surgical reimplantation had a 60% reduction in the risk of febrile UTI over a 5 year period of follow up. The authors concluded that it is uncertain whether identification and treatment of VUR confer any clinically important benefit. Although this was a well-performed study, many clinicians will disagree with the conclusions. In particular, a 60% reduction in the likelihood of febrile UTIs would likely be considered an important clinical achievement.

In an SR/MA on the effect of circumcision for prevention of UTI, Singh-Grewal and colleagues reviewed 12 studies including over 400 000 boys [6]. This included 1 RCT and 11 observational studies. The overall protective effect of circumcision was both clinically and statistically significant with an odds ratio of 0.13 (*P* < .0001). The effect was unaltered by study design. They estimated that in a general population, 111 circumcisions are required to prevent one UTI, due to a low incidence of UTI (1%). However, in certain subgroups of boys that are prone to UTI (such as those with VUR), the benefit of circumcision becomes more apparent. If the risk of recurrent UTI in patients with VUR is estimated to be between 10 and 30%, the number needed to treat will decrease to between 4 and 11.

This was a well-performed study without any major methodological flaws. Nonetheless, the quality of the included studies was variable. Methods of diagnosis of UTI were not uniform, follow ups were not similar and in some instances there was significant heterogeneity. Based on these findings, circumcision should be considered in the management of boys with VUR and UTI.

Elder and colleagues performed an MA on the success rate of endoscopic treatment of VUR [7]. They analyzed 63 publications encompassing 5527 patients and 8101 ureters. Only 3 studies were RCTs, with the rest being observational. All together, 5 different bulking agents were assessed, with only 6/63 (10%) of studies involving the use of Deflux, the most widely used agent today. They found out that the overall success rate of endoscopic treatment regardless of type agent used and grade of VUR is almost 75% with one injection. This can be improved to 85% with multiple treatments. High grade, neuropathic bladders, and duplicated ureters lowered the success rate. The reported rate of febrile UTI following treatment was less than 1% and cystitis occurred in 6% of cases. The paramount conclusion was that the success rate of endoscopic treatment approaches that of surgical reimplantation.

However, this study did not meet the standards for a well-done systematic review; the authors only interrogated the MEDLINE database plus a hand search of the references obtained as the basis for their literature search. An additional weakness is the possible heterogeneity of the studies in terms of their design, type of treatment, and length of followup. The authors did not address this issue by doing a test of heterogeneity.

Williams and colleagues performed an SR/MA to determine the efficacy of antibiotic prophylaxis for the prevention of UTI [8]. They included RCTs, comparing the effectiveness of antibiotics to each other or to placebo, in prevention of UTIs in children. They analyzed 8 studies, and as a subset evaluated the effect of antibiotics in prevention of UTI in children with VUR. Only 2 studies reported the outcomes separately for patients with and without VUR. These studies showed a 54% reduction in subsequent positive urine cultures. The authors detected significant heterogeneity amongst the 8 included studies. Moreover, the above outcome is not considered clinically important since most pediatric urologists would not treat asymptomatic bacteriuria.

Venhola et al. performed a meta-analysis of the efficacy of medical versus surgical treatment of reflux [9]. They used recurrence of UTI, renal damage, and renal growth as the outcomes. They screened 639 studies and included only 5 of them in the final analysis. They found that the bulk of studies in the literature on VUR is retrospective and poorly designed. Their results and conclusions were very similar to the SR done by Wheeler 2 years earlier. In summary, they did not show any evidence of superiority of surgical treatment in preventing UTI, scars, or abnormal growth. This SR/MA has several shortcomings. The search strategy was suboptimal. The authors failed to identify at least another 4 trials that other authors have reported on. They combined the results of different study design types (RCT and cohort). The latter shortcoming is critical: the design of a study is so important that even if different types of studies reveal similar results, combining them may be misleading. Although mentioned in the article, they failed to emphasize a clinically important finding: the advantage of surgery over medical treatment in reduction in the likelihood of pyelonephritis.

Probably the most thorough SR in the VUR literature is a recent study by Hodson et al. from the Cochrane Renal Group [10]. This is an update of their SR on the treatment of VUR published in 2004. They performed an extensive and systematic literature review and identified 11 randomized controlled trials involving 1148 children. The RCTs included 7 comparing surgical (open or endoscopic) versus medical treatment, 2 compared prophylaxis antibiotics with surveillance and 2 compared different endoscopic methods. Although there were a few methodological issues with some of the RCTs (e.g., blinding of the outcome assessors, intention to treat analysis), the overall quality of the 11 included was acceptable. The authors found that the risk of any UTI is not different between surgically and medically treated groups. However, surgical correction of VUR results in a 50% reduction in febrile UTI (RR 0.54, 95% CI 0.32–0.92). With a 5 year incidence of febrile UTI estimated at 20%, the authors estimated that the number needed to treat to prevent one event was 9. In other words, 9 reimplantations would be required to prevent one episode of pyelonephritis over a 5 year period. New or progressive renal damage had a similar incidence in the two groups. In two small RCTs (total 143 children) with short followup, the likelihood of UTI was similar in patient on prophylactic antibiotics versus no treatment. In RCTs, looking at different types of bulking agents silicone (Macroplastique) and Deflux had similar results in terms of VUR correction rate and recurrent UTI. In a small study, GAX 35 collagen has been shown to be inferior to GAX 65 in correcting VUR. The authors concluded that it is uncertain that surgical treatment of VUR leads to clinically important benefit.

## 4. DISCUSSION

VUR has been at the centre of many debates in pediatric urology for several decades, going through several paradigm shifts. Up to the late 1970’s and even the early 1980’s, VUR was considered a significant disease and was treated primarily with a variety of open surgeries. Subsequently, large RCTs such as the International and Birmingham Reflux Studies [11, 12] cast a shadow of doubt on surgical intervention as the management of first choice. These seminal studies were based on several assumptions:

1. VUR is a pathologic finding;
2. VUR facilitates UTI;
3. renal parenchymal infection may cause renal damage, hypertension, and renal insufficiency;
4. correction of reflux by surgery, or prevention of UTI with antibiotic prophylaxis until spontaneous resolution, prevents these unfavorable outcomes.

This resulted in failure of including another management strategy in these large studies, namely clinical surveillance. Nevertheless, a new perspective was generated: VUR can be managed medically and only selected patients will require surgery. These initial randomized studies also showed that surgical treatment reduces the likelihood of febrile UTI. Some authors would question the importance of this outcome without demonstrating a concomitant reduction in renal damage. However, one should not ignore the potential morbidity and even rare mortality associated with febrile UTI, especially in young children.

More recent findings have changed the landscape again. The fact that up to 50% of radiological renal defects could be congenital and not a consequence of UTI implies that VUR may be even less clinically important [13]. Studies have persistently failed to show a beneficial effect for treatment of VUR in reducing the risk of renal scarring, even when the incidence of febrile UTI is decreased.

The efficacy and safety of long-term antibiotics have also been questioned. A recent RCT by Garin et al. did not demonstrate any benefit from antibiotic versus surveillance in reducing febrile UTI in children with low and moderate grade VUR after one year of followup [14]. In addition long-term antibiotics may not be as harmless as we once thought [15, 16].

Another major advance is the introduction of a safe and effective bulking agent for endoscopic treatment of VUR, that is, Deflux. However, this method has never been compared to other management strategies in a prospective manner. Again our assumptions have preceded the evidence in adopting a treatment strategy.

Management of VUR also influences other important clinical decisions, such as when to image children with febrile UTI or siblings of patients with VUR [17]. Finally, the cost effectiveness and impact on quality of life for these investigations and treatments have not been assessed in prospective fashion.

We believe pediatric urologists should spearhead efforts to generate the high-level evidence guiding the management of VUR. The best way is to compare all the available strategies in a randomized controlled trial. Ideally, all important outcomes should be evaluated with adequate followup. This requires recruitment of several hundreds patients, randomizing them into 3 groups (surveillance, antibiotics, surgery) and following them for 4–5 years. Only with such a design will questions about recurrence of UTI and renal damage ever be answered. In addition effects of potential confounders such as sex, grade of VUR, mode of presentation and dysfunctional voiding can be evaluated. This will also provide an opportunity to compare the cost–benefit of each strategy.

Another major benefit of this ideal study is a better clarification of the magnitude of the clinical importance of VUR. For example, if surveillance is shown to be an acceptable long-term management, there is no reason to diagnose VUR, because it would not change our clinical approach. On the other hand, if active treatment is associated with a better outcome, one can conclude that VUR is a clinically significant phenomenon that requires diagnosis.

There are many barriers to performing an ideal RCT in children, especially those involving a surgical arm. Randomization between several divergent modalities is usually met with low parental acceptance. The requirement for a large sample size combined with long-term followup will considerably increase the cost, probably to millions of dollars. It is very difficult if not impossible to perform this type of studies in a single centre. Multicentre trials are inherently more expensive and difficult to run. Ethical issues may also impede the recruitment [18].

In spite of all the adversities, a few RCTs are underway to answer the above questions [19].

## 5. CONCLUSIONS

The quality of available studies regarding VUR is highly variable and in many cases suboptimal. Recent findings and advances in different aspects of VUR mandate a new look into our clinical management of this disorder. Ideally, a large multicentre randomized controlled trial should be done, including all available management strategies.

## ABBREVIATIONS

95% CI: 95% Confidence interval
DMSA: Dimercaptosuccinic acid
MA: Meta-analysis
QUOROM: Quality of reporting of meta-analysis
RCT: Randomized controlled trial
RR: Relative risk
SR: Systematic review
UTI: Urinary tact infection
VCUG: Voiding cysto-urethrogram
VUR: Vesicoureteral reflux
